# Techno–Economic Analysis of the Optimum Configuration for Supercritical Carbon Dioxide Cycles in Concentrating Solar Power Systems

**DOI:** 10.3390/e26020124

**Published:** 2024-01-31

**Authors:** Rosa P. Merchán, Luis F. González-Portillo, Javier Muñoz-Antón

**Affiliations:** 1 Department of Applied Physics and IUFFYM, University of Salamanca, 37008 Salamanca, Spain; rpmerchan@usal.es; 2 Departamento de Ingeniería Energética, Universidad Politécnica de Madrid, 28006 Madrid, Spain; lf.gonzalez@upm.es

**Keywords:** concentrating solar power, supercritical CO_2_, optimum configuration, techno–economic assessment, optimization

## Abstract

There is a general agreement among researchers that supercritical carbon dioxide (sCO_2_) cycles will be part of the next generation of thermal power plants, especially in concentrating solar power (CSP) plants. While certain studies focus on maximizing the efficiency of these cycles in the hope of achieving a reduction in electricity costs, it is important to note that this assumption does not always hold true. This work provides a comprehensive analysis of the differences between minimizing the cost and maximizing the efficiency for the most remarkable sCO_2_ cycles. The analysis considers the most important physical uncertainties surrounding CSP and sCO_2_ cycles, such as turbine inlet temperature, ambient temperature, pressure drop and turbomachinery efficiency. Moreover, the uncertainties related to cost are also analyzed, being divided into uncertainties of sCO_2_ component costs and uncertainties of heating costs. The CSP system with partial cooling (sometimes with reheating and sometimes without it) is the cheapest configuration in the analyzed cases. However, the differences in cost are generally below 5% (and sometimes neglectable), while the differences in efficiency are significantly larger and below 15%. Besides the much lower efficiency of systems with simple cycle, if the heating cost is low enough, their cost could be even lower than the cost of the system with partial cooling. Systems with recompression cycles could also achieve costs below systems with partial cooling if the design’s ambient temperature and the pressure drop are low.

## 1. Introduction

Supercritical CO_2_ (sCO_2_) Brayton cycles have a great potential for the cost reduction of thermal power plants due to their high efficiencies [1], but also due to their potential lower cost in comparison to steam cycles [2]. Steam cycles are more “cost effective and more thermally efficient” in current thermal power plants [3]. However, higher turbine inlet temperatures make sCO_2_ cycles more efficient and more cost-effective than steam cycles [4,5,6,7,8,9]. One of the potential uses for sCO_2_ cycles is its integration in high-temperature Concentrating Solar Power (CSP) plants [10,11], where the levelized cost of electricity (LCoE) reduction is expected to be between 15.6% and 67.7% in comparison to current CSP systems [4]. This cost reduction is essential in order to achieve further spread of commercial CSP plants [9].

Since there are several possible sCO_2_ cycle layouts, most studies have tried to identify the best configuration by analyzing thermal and/or exergetic efficiency [12]. A common outcome of these recent studies is that the recompression cycle presents higher efficiency than the simple one [10,13,14,15,16]. More specifically, White et al. concluded that the process efficiency could be moderately improved by including reheating and/or compressor intercooling [13]. Focusing on the integration with CSP plant types, two studies proved that, within a solar central receiver system, higher net specific work output and thermal efficiency are achieved in the recompression cycle, compared to the simple regenerative [14,15], to the precompression and to the split expansion cycle, due to the reduced compressor work [15]. Nevertheless, Neises et al. highlighted that their results do not tip the scales in the partial cooling cycle favor over the recompression one, but they recommended further studies of the partial cooling cycle with the aim of clarifying its advantages [17]. Furthermore, Padilla et al. demonstrated that the simple cycle could compete with the conventional regenerative steam Rankine cycle with respect to solar central tower systems [16].

On the other hand, the study of those sCO_2_ Brayton cycles, which are adapted for CSP systems, looking for the best layouts is not so usual from a techno–economic perspective, at least to the authors’ knowledge. This fact has been pointed out by Alfani et al., who indeed put the focus of their study on the trade-off between system cost and efficiency and highlighted the recompressed cycle with intercooling as the most promising cycle by means of a multi-objective genetic algorithm [18]. Other methods have been employed too, like the stochastic approach performed by Meybodi et al. [19]. However, other outcomes are found in the literature, like results from Marchionni et al.’s study, which showed that recompression configurations with reheating are linked with higher CAPEX per unit of electric power despite the higher efficiency [20]. Additionally, Cheang et al. concluded that the partial cooling cycle would constitute the “best” layout for CSP, looking at both maximum efficiency and minimum cost, though they are not competitive against current steam cycles [3]. Nonetheless, those analyses were made with a maximum temperature of 580 °C, which is not the ideal temperature for supercritical Brayton cycles. On the contrary, another study highlighted the advantages of sCO_2_ Brayton cycles over steam ones when integrating into CSP plants [4]. Moreover, Ho et al. concluded that, given a certain output power, higher efficiency sCO_2_ cycles are related to lower solar and power-block component costs [21]. Nevertheless, some of those studies [18,21] did not take into account the inherent uncertainties in cost and performance; only Crespi et al. have considered those cost uncertainties [22,23]. They found that sCO_2_ cycles are associated with similar, or even lower, LCoEs than traditional steam CSP plants, which supports sCO_2_ Brayton cycles as a viable option to improve CSP competitiveness [22,23].

When analyzing the different sCO_2_ layouts for CSP systems, the studies in the literature have focussed on different features. Neises et al. analyzed total recuperator conductance for the different cycles and how it influences cycle thermal efficiency [17]. Atif et al. studied how heat absorbed by a central receiver can induce turbine inlet temperature variability [14]. Turchi et al. assessed thermal efficiency by looking at turbine and compressor inlet temperatures [24]. The ambient temperature effect on cycle performance was evaluated by Neises et al. as a part of an off-design conditions test [25]. In addition, pressure drop has also been studied as a key parameter of those cycles [26].

This work provides a comprehensive analysis of the impact of the aforementioned features on the most remarkable cycles found in the literature. The objective is to identify the optimal configurations of sCO2 cycles that minimize the capital costs of CSP systems operating across various conditions. By exploring different ranges of conditions, the most cost-effective configurations for CSP systems are determined. The most significant physical uncertainties in these systems are addressed, from ambient temperature and turbine inlet temperature to pressure drop and turbomachinery efficiency. The effects of cost uncertainties are also analyzed, which have an enormous influence on the results, and which have not been previously analyzed according to the author’s knowledge. Although the main objective of this study is to optimize the CSP systems to minimize the capital cost, the systems are also optimized separately to maximize efficiency with the purpose of better understanding the importance of techno–economic analysis. Conventionally, some studies have maximized efficiency and specific power [27] since they were expected to minimize electricity costs [8]; however, this was not always true [28]. This work provides a comprehensive analysis of the differences between minimizing the cost and maximizing the efficiency for the most remarkable cycles found in the literature. The methodology employed is described in Section 2, where the model and its optimization are shown. Minimum-cost systems and maximum-efficiency systems are compared in Section 3. This section gathers all results with their corresponding discussion, and Section 4 is devoted to the main conclusions.

## 2. Methodology

This section describes the methodology followed in this study to optimize the CSP systems with sCO_2_ cycle. First, the system layouts are outlined, and the model used to simulate them is benchmarked against results from the literature. Then, the analyzed variables and their boundaries are explained. The last part of the section describes the optimization method used to obtain systems with minimum cost on the one hand and maximum efficiency on the other.

### 2.1. Cycle Configurations

**Figure 1:**
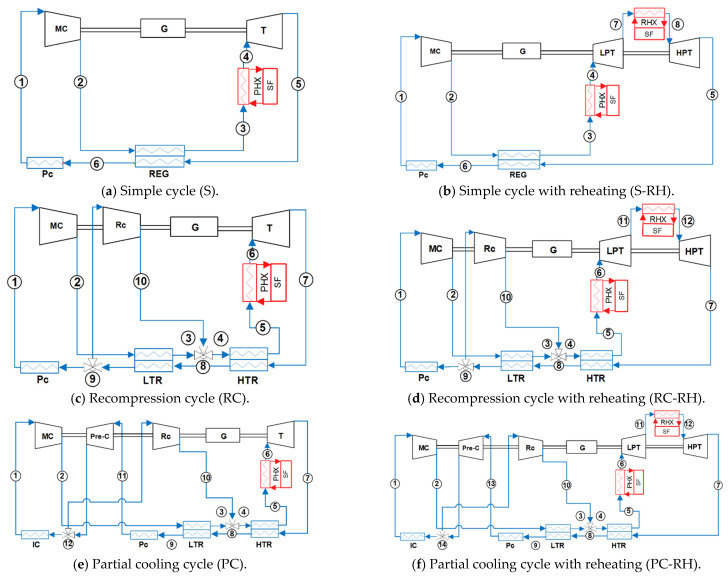
Component diagrams of the analyzed cycles.

**Figure 2:**
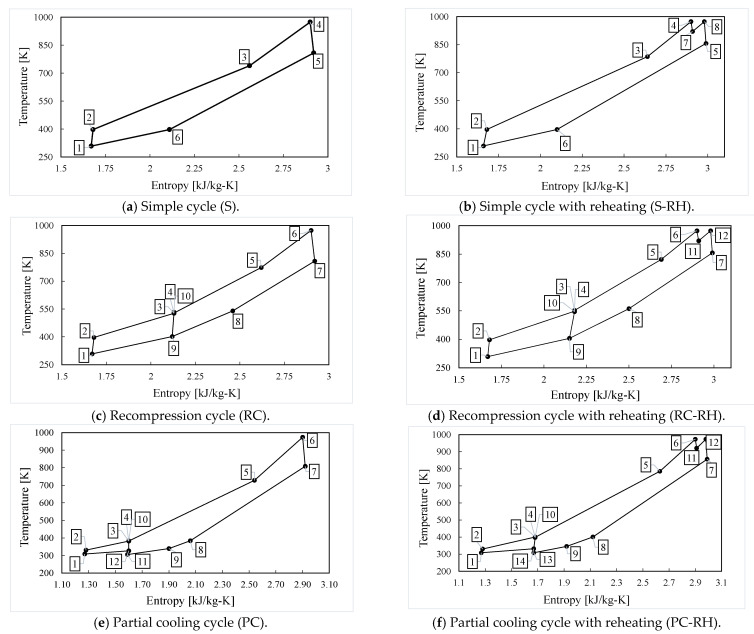
T-s diagrams of the analyzed cycles.

The following six configurations are considered in this comprehensive study, based on the most common cycles found in literature: simple, recompression and partial cooling layouts, all of them with and without reheating [3,4,11,13,14,15,16,17,20,29]. The components diagrams of these cycles are shown in Figure 1, meanwhile, Figure 2 shows examples of their T-s diagrams.

In the simple Brayton cycle (see Figure 1a and Figure 2a), the sCO_2_ enters a main compressor (MC), then the excess heat from the turbine outlet is absorbed by the sCO_2_ thanks to a regenerator (REG). Next, heat from the solar field (SF) is transferred to the fluid by means of a primary heat exchanger (PHX). After that heat absorption, the sCO_2_ is expanded in a turbine (T), and the excess heat is exchanged via the regenerator and via a precooler (Pc) with the ambient.

Regarding the recompression Brayton cycle (see Figure 1c and Figure 2c), a second compressor, the recompressor (Rc), is added to the layout when compared with the simple cycle. Additionally, the regenerator is divided into two different components: the low-temperature regenerator (LTR) and high-temperature regenerator (HTR). In this way, the hot outlet of the LTR is divided into two flows. One of the fractions of sCO_2_ (split ratio, SR) is compressed in the main compressor after being precooled, meanwhile, the other fraction is not precooled and is directly compressed in the recompressor. Then, the latter flow merges with the main flow of sCO_2_ after the cold outlet of the LTR and the whole flow exchanges heat in the HTR.

For the partial cooling Brayton cycle (see Figure 1e and Figure 2e), the same layout as in a recompression cycle is considered with the inclusion of a third compressor, the precompressor (Pre-C), and an intercooler (IC). In this case, the hot exit of the LTR is precooled and precompressed before being split into two flow fractions. One of them (split ratio, SR) is then cooled in the intercooler and compressed in the main compressor, reaching the cold inlet of the LTR, while the other one is directly recompressed and sent to the cold outlet of the LTR.

Concerning the reheating layouts (see Figure 1b,d,f and Figure 2b,d,f), two expansion processes are considered. Therefore, a low-pressure turbine (LPT) and a high-pressure turbine (HPT) are included in the cycle, while a reheater (RHX) is placed between these two turbines.

### 2.2. Model and Benchmark

The model has been built into EES software [30], version 10.836, which employs the REFPROP version 10.0 [31] database to obtain the thermodynamic properties of sCO_2_. The main part of the system is the sCO_2_ cycle. The mass and energy balance equations used to model the six cycle layouts are based on Padilla’s work [16]. The conductance (UA) of the heat exchangers was calculated by dividing the heat exchanger into a certain number of parts, see Table 1, “REG steps number”. Additionally, this discretization of the heat exchangers was useful for dealing with possible issues related to the temperature pinch point. The following main assumptions were considered for the development of the cycle model:

- the cycle operates in steady-state conditions;
- pressure drops are considered in each component as a percentage of the working pressure;
- compressors and turbines work in adiabatic conditions.

**Figure 3:**
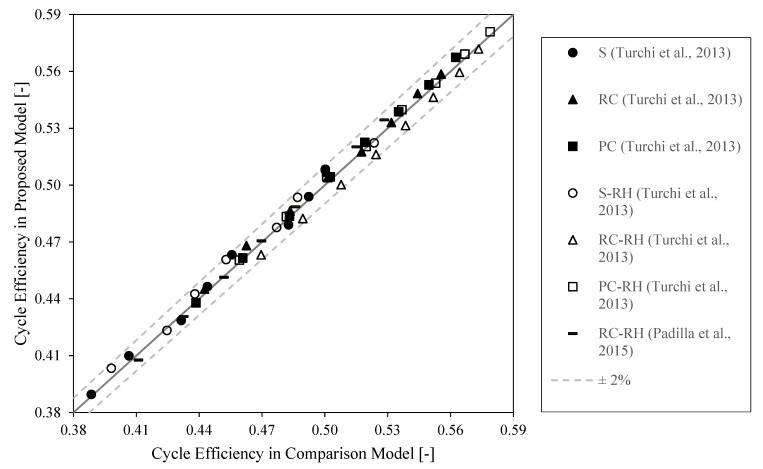
Parity plot between the literature results [16,24] and proposed model.

The cycle energy model has been benchmarked by comparing the results obtained with our model against results from the literature [16,24]. Figure 3 shows the comparison of the efficiencies obtained for different cycle layouts and different turbine inlet temperatures. The cycle efficiency has been maximized in our model, as in Turchi et al. [24,32]. All six proposed cycles showed a relative deviation lower than 2% [7,15].

In this study, cycle features were divided among parameters that were fixed, variables that were optimized, and variables that were aimed to be analyzed, as is shown in Table 1. High pressure, power output, and minimum pinch point temperature were set to values commonly used in the literature [24]. The range of values analyzed in the analyzed variables is further explained in Section 2.3, and the optimization process for the optimized variables is in Section 2.4.

A cost model has been included in the code along with the energy model for the adequate economic evaluation of the cycles (see Appendix A). The cost of the following components is calculated according to the correlations from Weiland et al. [33]: recuperators, direct air coolers, turbomachinery, gearboxes, generators, and motors. The cost of the sCO_2_ piping is estimated by adding an extra 10% to the cycle cost. The maximum temperature of each component, as well as a parameter related to the scale of the corresponding component, are introduced as inputs of these correlations. In this way, the cost of each cycle component is computed by means of the cost correlations and scaled by the cycle power. Although the turbine cost correlation is valid until 730 °C, it is employed as an approximation for the study of higher temperatures.

Solar field and primary heat exchanger costs are introduced in the system model as a unique parameter, “heating cost”. “Heating cost” integrates the cost uncertainties of these systems under one parameter. Since the cost of the solar field and the primary heat exchangers have high uncertainties (the type of receiver, type of heat exchanger, solar multiple…), the use of “heating cost” allows us to analyze the best cycle layout under different cost conditions in a simple way, which is one on the main novelties of this work.

### 2.3. Variables to Analyze

The best configuration of system layouts is calculated by optimizing the optimized variables from Table 1. These optimizations are made for different values of the following parameters: ambient temperature, turbine inlet temperature, heating cost, pressure drop, and turbomachinery efficiency. Table 1 gathers the design point and the range bounds of these parameters.

The ambient temperature mainly depends on the weather conditions. This study sets the conditions for the design point. Although weather conditions change over time, previous studies suggest that cycle performance at any off-design ambient temperature conditions are similar to the respective on-design behavior [25]. This study analyzes the CSP systems for different ambient temperatures between 30 °C and 40 °C, which are the temperatures commonly analyzed in the literature [17,21,24,25,33,34,35,36]. Moreover, the range between 20 °C to 30 °C is also analyzed to generalize the results by covering wet-cooling applications too [37]. The compressor inlet temperature will be calculated to reduce the plant-specific cost, setting a minimum of 32 °C [37] in order to keep in the supercritical region.

The compressor inlet temperature, which directly depends on the ambient temperature, is optimized for every system. This optimization found that, for ambient temperatures of 30–40 °C, the optimum compressor inlet temperature obtained to minimize the cost and maximize the efficiency was always 5 K greater than the ambient temperature. This result is because of the considered restrictions of Table 1: the minimum pinch point temperature is set to 5 K. In this work, a minimum pinch point temperature difference of 5 K is enforced in the heat exchangers, following the approach of Turchi et al. [24]. Ambient and compressor inlet temperatures are linked by a minimum pinch point of 5 K. The compressor inlet temperature has been optimized since it can be changed according to the design of the component, however, the ambient temperature cannot, thus, it is considered as an analyzed variable that depends on the weather conditions.

Regarding the turbine inlet temperature, the analysis of the range 500–900 °C is common in nuclear reactors [4], and very similar values are used for the analysis in CSP applications, 500–850 °C [16,24,26,38]. Achieving these turbine inlet temperatures is subject to reaching higher temperatures in the heat transfer fluid. The highest temperatures could be achieved with particle receivers [21] or liquid metals [39], and the lowest temperature with current commercial molten salts [40].

The temperatures achieved in the solar receiver highly affect the cost of the solar field and the heat exchanger. These two costs are analyzed together in this study under the parameter heating cost. This cost not only depends on the temperatures achieved in the system, but also on the solar field size (commonly analyzed by the solar multiple). The heating cost with current CSP costs could vary between 1535 USD/kW_t_ and 2329 USD/kW_t_ for solar multiples 2 and 3, respectively [41]. Although these costs could be greatly reduced to 818 USD/kW_t_ and 1253 USD/kW_t_, respectively, if the Sunshot goals are achieved [41]. Particle-based systems for the next generation of CSP plants with high solar multiple show heating costs of 1600 USD/kW_t_ [42]. These costs increase to 1944 USD/kWt without the heat exchanger in the study from Cheang et al. [3]. The system advisor model [43] suggests 1249 USD/kWt in the system with molten salts solar tower without including land and primary heat exchanger cost. After the literature review, the range of values selected for the analysis is 1000–2000 USD/kW_t_, and the design value 1500 USD/kW_t_.

The turbomachinery efficiency also has several uncertainties. Very high values around 0.9 are commonly used in the literature [17,44]. However, DOE recently suggested values of 0.8 and 0.87 for compressor and turbine efficiency, respectively [45]. The reality is that there is a lack of experimental tests to corroborate these values. Thus, this study analyzes turbomachinery efficiencies from 0.7 to consider worse scenarios than expected, which could happen for small powers.

The pressure drop is difficult to estimate since it depends on several factors. Vendors estimate pressure drops in sCO_2_ recuperators from 0.7 to 4 bars, and from 0.5 to 1.5 bars on the sCO_2_ side of the dry cooler [33]. Seo et al. have modelized a sCO_2_ heat exchanger whose maximum outlet pressure is 200 bar at operating conditions and with a 0.75% pressure drop [46]. Additionally, both Siddiqui et al. [26] and NETL (National Energy Technology Laboratory) [47] have accounted for the effect of pressure drops in the cycle within the interval 0–4%. Reznicek measured experimentally the pressure drop for sCO_2_ recompression Brayton cycles, getting a value of 2.5% for LTR hot-side as baseline and bounds of 0.3–6.6% [48]. Moreover, Padilla et al. considered pressure drops of 1%, 2.5%, and 5% in the solar central receiver and fixed a pressure drop in heat exchangers of 82.74 kPa [16]. Finally, Zhang et al. tested the impact of different pressure drop fractions (0–3%), concluding that they have a significant effect on energy performance [49]. Thus, following the mentioned approaches, a design pressure drop of 1.5% is selected, with lower and upper bounds of 0% and 4.5%, respectively.

### 2.4. Cycle Optimization

The objective of the study is to find the minimum-cost system layout under different conditions. Thus, the systems are optimized to obtain the configuration with minimum electricity specific cost (in USD/kW_e_). The configurations needed to achieve the maximum cycle efficiency are obtained for comparison. The variables for each optimization are regenerator effectiveness, pressure ratio, compressor inlet temperature, split ratio (for recompression and partial cooling cycles), pressure at the precompressor outlet (if partial cooling cycles), and pressure at the reheater inlet (in the case of reheating cycles).

The optimization method used is the “Genetic method” from EES, which uses “a robust optimization algorithm that is designed to reliably locate a global optimum even in the presence of local optima” [30]. It is essential that the algorithm used for this type of optimization can find local optima. The reason is that small changes close to the critical point can lead to very different tendencies [50] that mislead the optimizer. Other simpler algorithms that were unable to find local optima showed much worse results. Even using the “Genetic method”, several iterations were sometimes needed to find the optimum values.

The procedure considers as input variables the analyzed variables in Table 1 and the optimized solution refers to the values of “optimized variables” of Table 1 that reach the optimum result.

## 3. Results

This section analyzes the impact of the main variables affecting the optimum cost of the CSP system. The first sections analyze the effect of the variables from Table 1, and the last section analyzes the uncertainty of cycle component costs. The objective is to find the cycle configuration with the minimum specific cost in the different cases. The differences between maximizing efficiency and minimizing cost are used to understand the advantages and disadvantages of each configuration in terms of cost. The results shown in this section are cycle efficiencies and system costs. Conductances and pressure ratios linked to those results can be found in Appendix B.

### 3.1. Turbine Inlet Temperature

**Figure 4:**
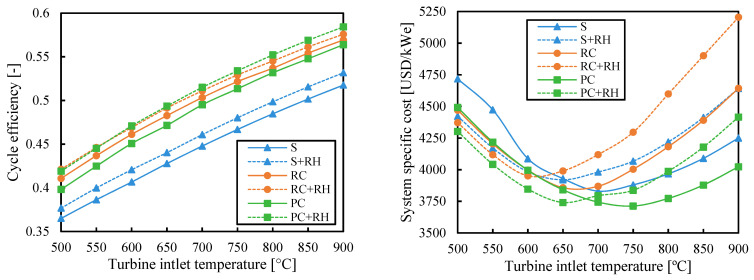
Cycle efficiency and system-specific cost as a function of turbine inlet temperature when the efficiency is maximized. Legend: S (simple), RC (recompression), PC (partial cooling), and RH (reheating).

Figure 4 shows cycle efficiency and specific cost of the analyzed cycles as a function of turbine inlet temperature when the efficiency is maximized. The efficiency of all cycles increased with the turbine inlet temperature (as did the Carnot efficiency η_C_ = 1 − T_cold_/T_hot_). The reheating (RH) is useful to slightly increase the efficiency of all configurations. As a result, the partial cooling cycle with reheating (PC-RH) was the most efficient layout.

Previous studies [16,18] have chosen the recompression cycle as the cycle with the greatest potential by assuming that its high efficiency would lead to a lower specific cost. However, Figure 4 suggests that this may not be true. If the system costs of the cycle configurations obtained for maximizing the efficiency are calculated, the recompression cycle with reheating (RC-RH) is shown to be the most expensive for medium-high values of turbine inlet temperature. The large heat exchangers needed to achieve high efficiencies in recompression cycles are the main contributors to this high cost (see Appendix B.1). Since the conductance (used to measure the heat exchanger size) is not limited to maximize efficiency, some systems, such as the one with the recompression cycle, can reach very high costs.

Figure 4 also shows that the reheating (RH) can increase the cycle-specific cost despite increasing the efficiency when the turbine inlet temperature is high. The main reason is the higher cost of the HTR when there is reheating. The HTR works at higher temperatures when there is reheating, which highly increases the cost, especially at high temperatures. So when the turbine inlet temperature increases, the high cost of the regenerator outweighs the benefit of the higher efficiency of reheating. Another reason for the higher cost of reheating is the higher cost of two turbines instead of one. In the cases of low turbine inlet temperature, this greater cost is outweighed by the higher efficiency, and the cycle with reheating is cheaper.

Component costs increase substantially when its maximum temperature is above 550 °C [33]. These cost increments overcome the benefit of the higher efficiencies at high turbine inlet temperatures and lead to higher system-specific costs. On the other hand, systems with low turbine inlet temperatures can use cheaper materials, but the smaller cycle efficiencies involve greater system-specific costs due to the bigger (and so more expensive) solar fields that are needed. As a result, there is a minimum system-specific cost for each layout found at turbine inlet temperatures between 650 °C and 750 °C.

**Figure 5:**
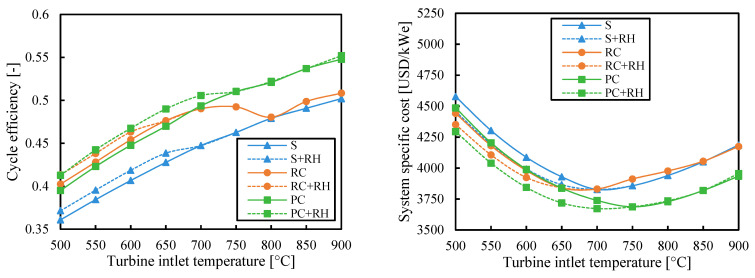
Cycle efficiency and system-specific cost as a function of turbine inlet temperature when the system cost is minimized. Legend: S (simple), RC (recompression), PC (partial cooling), and RH (reheating).

Figure 5 shows the results obtained when the main optimization objective is to minimize the system-specific cost. The system with simple cycle and reheating (S-RH) achieves lower costs than the simple system without reheating (S) when the turbine inlet temperature is less than 700 °C. However, the optimum configuration of the simple cycle with reheating (S-RH) deletes the reheating at higher turbine inlet temperatures, which makes it a simple cycle without reheating (S). The same occurs for the recompression (RC) and partial cooling (PC) cycles. The lines of systems with and without reheating overlap at high temperatures because reheating cannot reduce the system cost.

The lines of simple (S) and recompression (RC) systems overlapped at high turbine inlet temperatures. In these cases, the minimum-cost configuration of the system with a recompression cycle did not include a secondary compressor (i.e., all the fluid went through the main compressor), which made the recompression (RC) cycle a simple (S) cycle. This means that the system with recompression (RC) cycle at high turbine inlet temperatures achieved low specific costs at the expense of reducing the cycle efficiency up to the simple (S) cycle values.

The specific costs obtained for the system with recompression (RC) cycle by minimizing the cost (Figure 5) were significantly lower than those obtained when maximizing the efficiency (Figure 4) at high turbine inlet temperatures. The minimum-cost configuration was quite different from the configuration obtained for maximizing efficiency (see Appendix B.1). The costs obtained for the system with partial cooling (PC) and for the simple system (S) did not change as much as in the system with the recompression (RC) cycle from Figure 4 to Figure 5. The cycle configuration in these systems was more similar when the efficiency was maximized and when the cost was minimized.

Figure 5 shows that the minimum cost is achieved by the partial cooling cycle: with reheating for low temperatures and without it for high temperatures. The cost difference between the simple (S) cycle and the partial cooling configurations is in the range of 160–285 USD/kW_e_, which represents a difference of 4.3–6.6% (where the smallest difference corresponds to the turbine inlet temperature of 700 °C). These differences are much lower than the expected ones when the maximum cycle efficiencies were compared (Figure 4). In this case, the relative difference in efficiency between the maximum efficiency cycle (partial cooling cycle with reheating, PC-RH) and the minimum efficiency cycle (simple cycle, S) was between 11.4% and 13.7% (which corresponds to an absolute difference of 5.4–6.8%).

### 3.2. Ambient Temperature

**Figure 6:**
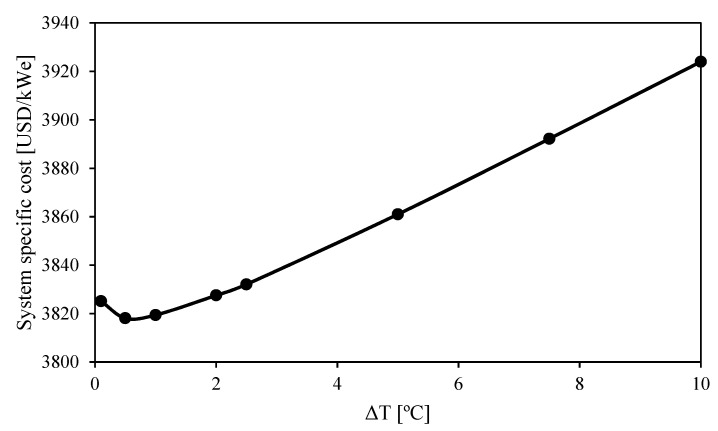
System-specific cost as a function of the temperature difference between compressor inlet temperature and ambient temperature when the system cost is minimized for the simple cycle without reheating.

A first analysis for evaluating the relationship between ambient temperature and compressor inlet temperature was performed. In this case, ambient temperature was fixed at 35 °C, while compressor inlet temperature was varied in such a way that the difference between them changed in the range [0.1–10] °C. The system-specific cost of a simple cycle without reheating was minimized by optimizing pressure ratio and regenerator effectiveness. Figure 6 shows that a decrease in this temperature difference led to a decrease in the system-specific cost, except when the temperature difference was really small. Hence, the election of the compressor inlet temperature was made based on the minimum pinch point temperature difference (set to 5 °C, see Table 1).

**Figure 7:**
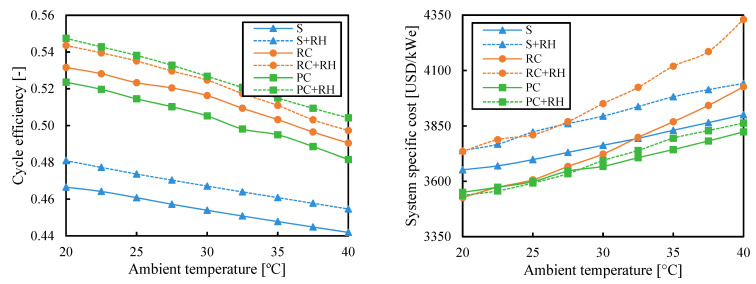
Cycle efficiency and system-specific cost as a function of ambient temperature when the efficiency is maximized. Legend: S (simple), RC (recompression), PC (partial cooling), and RH (reheating).

Figure 7 shows cycle efficiency and system-specific cost of the analyzed systems as a function of ambient temperature when the efficiency is maximized. The lower the ambient temperature, the higher the efficiency (as does the Carnot efficiency η_C_ = 1 − T_cold_/T_hot_). The recompression (RC) cycle achieved the highest increase in efficiency by lowering the ambient temperature from 40 °C to 30 °C, with a relative increase of 5% (absolute increase of 2.6%). These values are similar to the ones obtained by the partial cooling (PC), 4.2% relative increase, but far away from the 2.6% relative increase obtained with the simple (S) cycle. Lower ambient temperatures involved greater proximity to the critical point, which led to higher irreversibilities inside the regenerator that partial cooling and recompression cycles can better compensate.

The partial cooling cycle with reheating (PC-RH) was the most efficient when the efficiency was maximized and also the least expensive for low ambient temperatures. However, for high ambient temperatures, removing the reheating reduces the system costs. The second most efficient layout was the recompression cycle with reheating (RC-RH), but it was also the most expensive. Adding reheating clearly increased the efficiency for all cycles. But this raise was more significant for the partial cooling (PC) layout, which can achieve efficiencies around 4% higher, in relative terms, if the reheating is considered.

**Figure 8:**
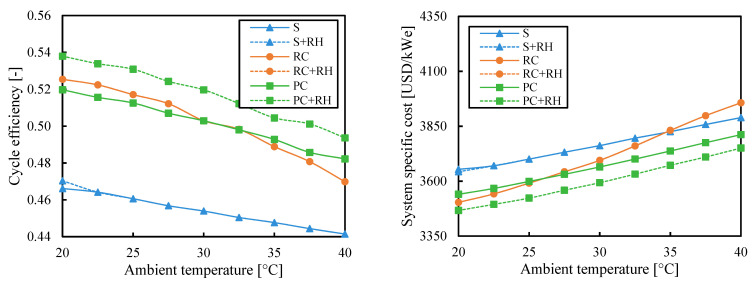
Cycle efficiency and system-specific cost as a function of ambient temperature when the system cost is minimized. Legend: S (simple), RC (recompression), PC (partial cooling), and RH (reheating).

If the objective is to minimize cost, Figure 8 shows that the partial cooling with reheating (PC-RH) cycle can be the cheapest both at low ambient temperatures and at high temperatures. The system with the simple (S) cycle was the most expensive for ambient temperatures lower than 35 °C, while the recompression (RC) one surpassed it. The cost of the recompression cycle (RC) grew faster at high ambient temperatures.

The simple cycles with and without reheating (S-RH and S) overlapped in the whole range of ambient temperatures (with the only exception of the lowest ambient temperature: 20 °C) in the same way as the recompression cycle with and without reheating (RC-RH and RC). The partial cooling cycle (PC) was the only configuration that could take the benefit of reheating. This occurred due to the 700 °C used as the turbine inlet temperature for this analysis. While the turbine inlet temperature affected the benefit of reheating (as shown in Figure 5), the ambient temperature did not.

### 3.3. Heating Cost

**Figure 9:**
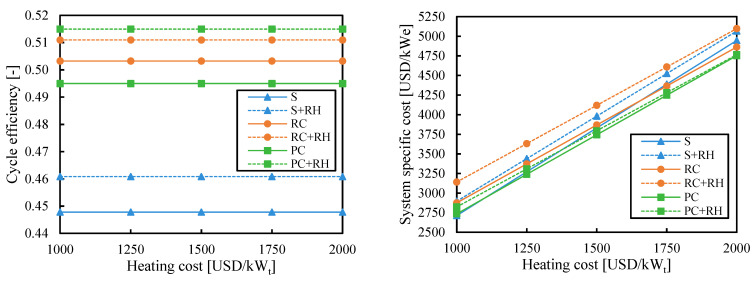
Cycle efficiency and system specific cost as a function of heating cost when the efficiency is maximized. Legend: S (simple), RC (recompression), PC (partial cooling), and RH (reheating).

Since the heating cost in CSP (composed of the solar field and primary heat exchanger) is the greatest cost of the system [18], the objective of increasing the cycle efficiency has always been to reduce the effect of the heating cost on the system cost. However, previous figures have shown that the highest efficiency cycle configuration may not be the cheapest one. Figure 9 analyzes the influence of the heating cost when the efficiency is maximized. The heating cost had no impact on the cycle efficiency since the former was not considered in the optimization to maximize efficiency. However, it had a big impact on the system cost. For example, systems with a simple (S) cycle showed the lowest costs when the heating cost was 1000 USD/kW_t_, but they were more expensive than systems with partial cooling (PC) when the heating cost was 2000 USD/kW_t_.

**Figure 10:**
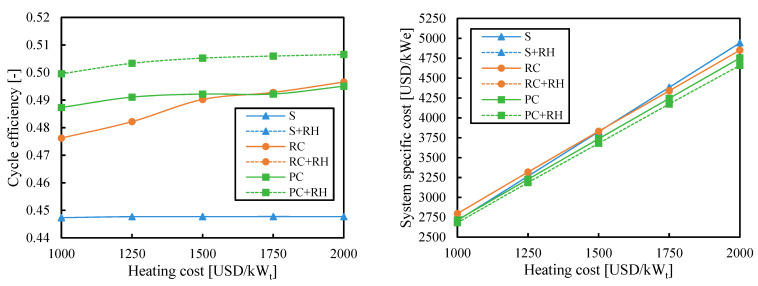
Cycle efficiency and system-specific cost as a function of heating cost when the system cost is minimized. Legend: S (simple), RC (recompression), PC (partial cooling), and RH (reheating).

Figure 10 shows the influence of the heating cost when the system cost was minimized. The efficiency of the simple (S) cycle was the same as in Figure 9 since minimizing cost and maximizing efficiency led to the same cycle configuration. The efficiency of cycles with partial cooling (PC and PC + RH) was slightly reduced from the results obtained when the efficiency was maximized (Figure 9). Moreover, the cycle efficiency of these cycles slightly increased with the heating cost since increasing the efficiency was more relevant when the heating was more expensive. This effect was even more pronounced in the cycle with recompression (RC).

Although the most economic cycle was the one with partial cooling and reheating, the difference in cost with the simple cycle was almost negligible (28 USD/kW_e_, i.e., 1%) when the heating cost was small. This difference increased up to 283 USD/kW_e_ (i.e., 6%) when the heating cost was high. More complex systems take more advantage of their high efficiency when the heating is more expensive.

### 3.4. Pressure Drop

The cycle efficiency decreased with the pressure drop and the specific cost increased. However, the pressure drop is a value with high variability in the analysis of power cycles. This value depends on the heat exchangers design and the connections between the different components. This study gives a specific relative pressure drop to each heat exchanger. This is a way to penalize the cycle configurations with a greater number of components.

**Figure 11:**
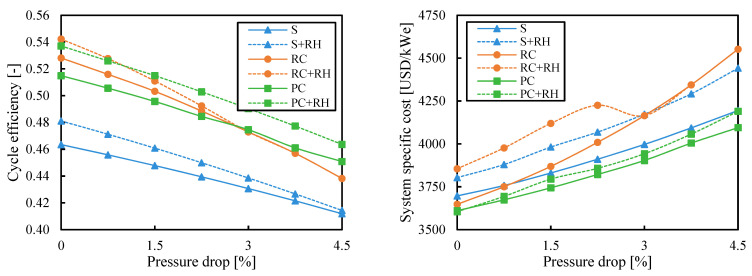
Cycle efficiency and system-specific cost as a function of pressure drop when the efficiency is maximized. Legend: S (simple), RC (recompression), PC (partial cooling), and RH (reheating).

**Figure 12:**
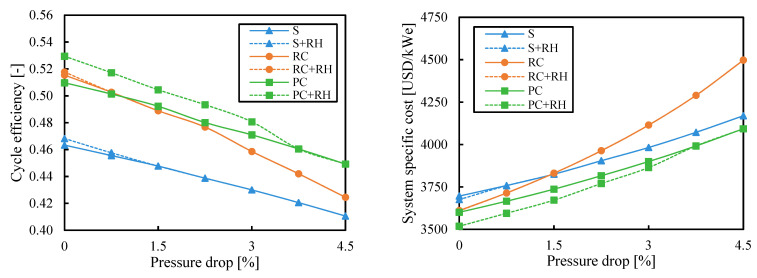
Cycle efficiency and system-specific cost as a function of pressure drop when the system cost is minimized. Legend: S (simple), RC (recompression), PC (partial cooling), and RH (reheating).

Figure 11 and Figure 12 show the results obtained when the cycle efficiency was maximized and when the cost was minimized, respectively, for different values of pressure drop. The pressure drop had a bigger impact on the recompression cycle. The simple cycle could better deal with pressure drop due to the smaller number of components, and the cycles with partial cooling were less affected by pressure drop because of the higher pressure ratios.

Note that these results were obtained for an ambient temperature of 35 °C. However, if the ambient temperature was reduced to 25 °C or 30 °C, the cost of the recompression cycle would be closer to the configuration with partial cooling (see Figure 8) or even below if the pressure drops were kept below 1.5%. In this case, the recompression cycle could be preferred over the cycles with precooling and reheating to reduce the system complexity.

### 3.5. Turbomachinery Efficiency

Cycle efficiency increased with the turbomachinery efficiency and the system specific cost decreased. However, it is interesting to compare the influence of this variable on the different cycle layouts. Although the most common values for compressor and turbine efficiency used to simulate sCO_2_ cycles were 0.89 and 0.93, respectively, [44] they could be lower in real systems.

**Figure 13:**
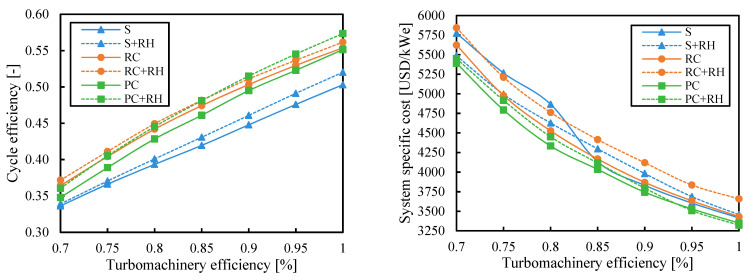
Cycle efficiency and system-specific cost as a function of turbomachinery efficiency when the efficiency is maximized. Legend: S (simple), RC (recompression), PC (partial cooling), and RH (reheating).

**Figure 14:**
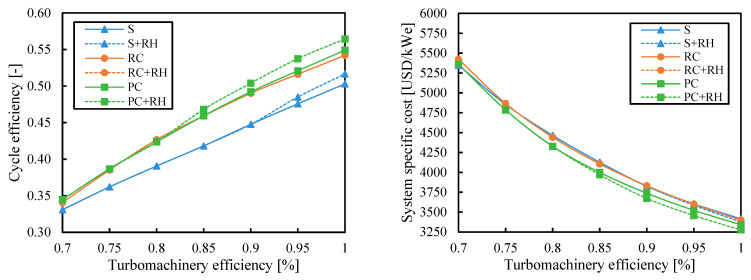
Cycle efficiency and system-specific cost as a function of turbomachinery efficiency when the system cost is minimized. Legend: S (simple), RC (recompression), PC (partial cooling), and RH (reheating).

Figure 13 and Figure 14 show the results obtained when the cycle efficiency was maximized and when the cost was minimized, respectively. Figure 13 shows that the more complex systems obtained a higher benefit in efficiency with higher turbomachinery efficiencies. The highest cycle efficiencies were achieved by the partial cooling cycle with reheating for turbomachinery efficiency higher than 0.85. On the other hand, for lower turbomachinery efficiencies, the recompression cycle with reheating reached better cycle efficiencies.

The cost differences between the systems were small at low turbomachinery efficiencies when the cost was minimized in Figure 14. In the range of efficiencies expected for sCO_2_ turbomachines (0.85 to 0.95), the cost difference between the most expensive and cheapest system was 147–157 USD/kW_e_ (3.8–4.1%). However, if the efficiency was 0.7, this difference would be almost negligible, and the simple cycle could be selected as the most economical choice. For turbomachinery efficiencies lower than 0.8, minimum system costs were found for the partial cooling (PC) cycle. Then, at higher turbomachinery efficiencies, adding the reheating to the partial cooling decreased the system cost by up to 1.9% in relative terms.

### 3.6. Cost Uncertainties

Any techno–economic analysis is full of uncertainties that are difficult to address. Some of the uncertainties related to sCO_2_ power cycles, such as the heat source cost, have been analyzed in this paper. However, there is a highly relevant uncertainty that has not been analyzed yet: the uncertainty of the cost correlations. Table 2 shows this uncertainty for each cycle component [33]. These uncertainties are used to obtain the minimum cost configuration of each layout in the following scenarios at the reference conditions from Table 1:

- reference case: no uncertainty used;
- cheap HX and expensive TM: lower bounds applied to heat exchangers (HX) and upper bounds applied to turbomachinery (TM);
- expensive HX and cheap TM: upper uncertainties applied to heat exchangers (HX) and lower uncertainty applied to turbomachinery (TM);
- expensive HX and expensive TM: upper uncertainties applied to heat exchangers (HX) and turbomachinery (TM);
- cheap HX and cheap TM: lower uncertainties applied to heat exchangers (HX) and turbomachinery (TM).

**Figure 15:**
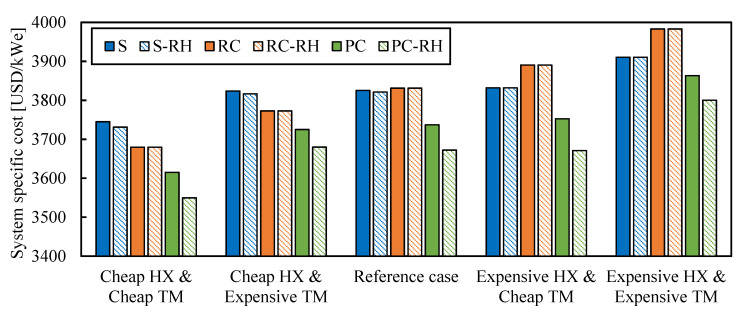
System-specific cost of studied cycles for different scenarios that account for the cost uncertainties. Legend: S (simple), RC (recompression), PC (partial cooling), and RH (reheating).

Figure 15 compares the specific cost of the analyzed systems for the different scenarios. The minimum cost layout is the system with partial cooling and reheating (PC-RH) in all the scenarios: 2.5–6.0% higher costs were found for the recompression (RC) cycle and 2.9–5.5% for the simple (S) cycle, depending on the case scenario. Those differences were big enough to consider the partial cooling cycle with reheating (PC-RH) as the recommended option for the first generation of sCO_2_ cycles with the reference conditions from Table 1.

Although the minimum cost configuration for the reference case was the one with partial cooling, it is interesting to observe the differences between the simple and recompression cycles for the different scenarios. As previously mentioned, these two configurations could achieve lower costs under different conditions. For example, the simple cycle would benefit more from low heating costs and the recompression cycle from low ambient temperatures. For the scenarios with cheap heat exchangers, the recompression (RC) cycle gives lower costs than the simple (S) cycle. However, in the case of scenarios with expensive heat exchangers, the simple (S) cycle performs better than the recompression (RC) regarding system costs.

## 4. Conclusions

This study shows the great utility of techno–economic analysis in terms of capital costs in CSP systems with sCO_2_ cycles. Although maximizing the cycle efficiency can show the potential of the cycle, the optimum cycle configuration can substantially change if the objective is to minimize the system cost. Most of the time, this more economical configuration is obtained at the expense of reducing the cycle efficiency, but it gives a better guide to choosing the best system configuration, which depends on the boundary conditions. Hence, this study also provides a comprehensive analysis of the differences between minimizing the cost and maximizing the efficiency for the most remarkable sCO_2_ cycles.

The CSP system with partial cooling (sometimes with reheating and sometimes without it) is the cheapest configuration in the shown cases. Nevertheless, the differences in cost are generally below 5% (and sometimes neglectable), while the differences in efficiency are significantly larger and below 15%. The optimum turbine inlet temperature is 700 °C, which perfectly fits the next generation of solar receivers that can achieve temperatures above 720 °C. In case the receiver cannot reach these temperatures, the most economical layout would keep being the partial cooling cycle with reheating. Although it would make no sense to increase the turbine inlet temperature above 700 °C because the system cost would grow, it is remarkable that then reheating would not be useful to reduce the system cost.

Although the cheapest configuration resulting from this paper contains cycles with partial cooling, systems with simple cycles could achieve very similar or even lower costs if the heating cost is low or if the turbomachinery efficiency is also low. If the heating costs were as low as 1000 USD/kW_t_, (which could happen in CSP systems with small solar multiple), the system cost with simple cycles would be very similar to the ones obtained with partial cooling but with a fairly simpler cycle. If the turbomachinery efficiency was as low as 0.7, the costs of both systems would also be very similar. If the two conditions of low heating cost and low turbomachinery efficiency were combined, the cost of systems with simple cycles could be even lower than the cost of the systems with partial cooling. The Solar Power Gen3 Demonstration Roadmap from the National Renewable Energy Laboratory (NREL) [51] states that the operation of high-temperature CSP plants should be performed with the help of systems that are as simple as possible [52]. Therefore, in the search of that simplicity, the sCO_2_ simple Brayton configuration would be preferred in those cases when really similar costs are found.

Systems with recompression cycles could also achieve very similar or even lower costs than systems with partial cooling if the design ambient temperature and the pressure drop were reduced. The ambient temperature is mainly given by the weather conditions. For low ambient temperatures such as 20 °C, the recompression cycle is only 1% more expensive than the partial cooling cycle with reheating, which could tip the scales in favor of the recompression due to its simpler layout. Moreover, small pressure drops would benefit more from the recompression cycle than the partial cooling cycle. Thus, a combination of design ambient temperatures below 30 °C and low-pressure drop would lead to the recompression cycle to be the most economical one.

In summary, the results shown in this study can help guide the choice of the most economical cycle layout, not only for CSP systems but also for other thermal power plants. A deeper analysis of the pressure drop as a function of heat exchanger size could improve the uncertainty of the results shown in this paper. However, the tendencies will probably be still the same.

## Figures and Tables

**Table 1:** The parameters of the energy model divided between fixed, optimized, and analyzed.

Fixed Parameter	Value	Optimized Variable	Value
High pressure	25 MPa	REG/global effectiveness	Optimized
Minimum pinch point temperature difference	5 K	Pressure ratio	Optimized
REG steps number	20	Compressor inlet temperature	Optimized
HTR/LTR/IC steps number	10	Split ratio (RC and PC)	Optimized
Pc steps number	5	Pressure at the outlet of Pre-C (PC)	Optimized
Cycle power	50 MW		
		Pressure at RHX inlet (RH)	Optimized
**Analyzed Variable**	**Reference Value**	**Lower Bound**	**Upper Bound**
Ambient temperature	35 °C	20 °C	40 °C
Turbine inlet temperature	700 °C	500 °C	900 °C
Heating cost	1500 USD/kW_t_	1000 USD/kW_t_	2000 USD/kW_t_
Pressure drop	1.5%	0%	4.5%
Turbomachinery efficiency	0.90	0.7	1

**Table 2:** Uncertainties of the cycle component costs [33].

Component	Component Type	Lower Bounds	Upper Bounds
Recuperator	Heat exchanger	−31%	38%
Cooler	Heat exchanger	−25%	28%
Turbine	Turbomachine	−25%	30%
Compressor	Turbomachine	−39%	48%

**Figure A1:**
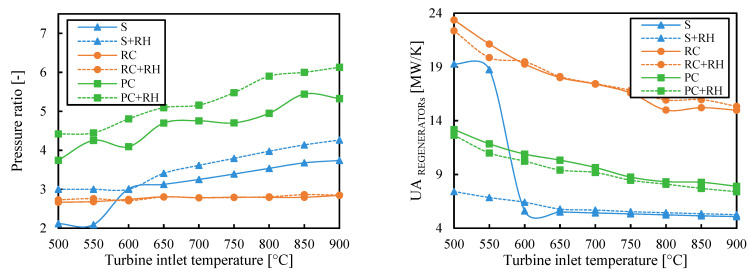
Pressure ratio and regenerator conductance used to maximize the cycle efficiency shown in Figure 4.

**Figure A2:**
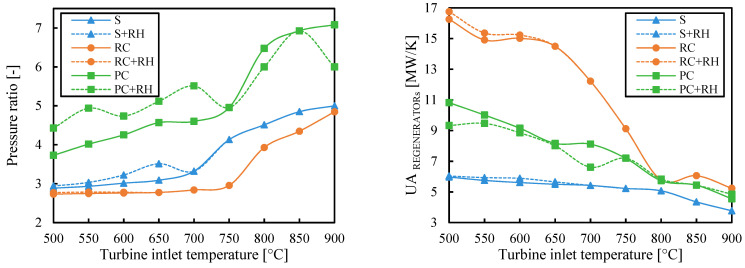
Pressure ratio and regenerator conductance used to minimize the electricity-specific cost shown in Figure 5.

**Figure A3:**
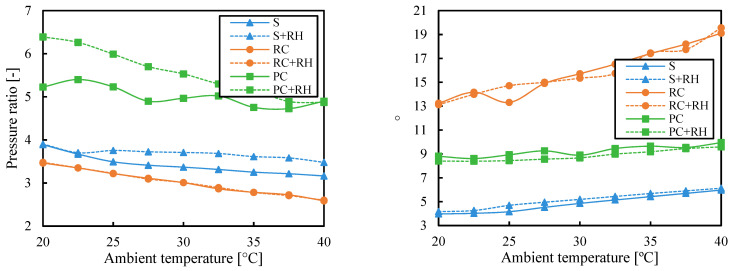
Pressure ratio and regenerator conductance used to maximize the cycle efficiency shown in Figure 7.

**Figure A4:**
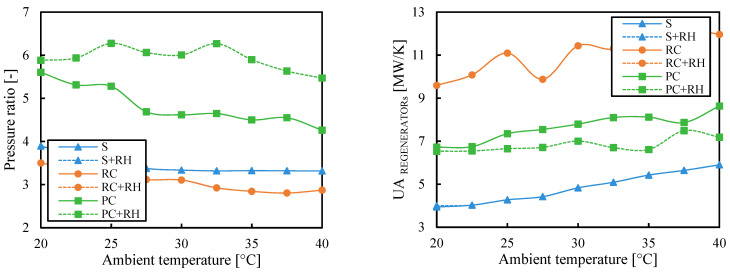
Pressure ratio and regenerator conductance used to minimize the electricity-specific cost shown in Figure 8.

**Figure A5:**
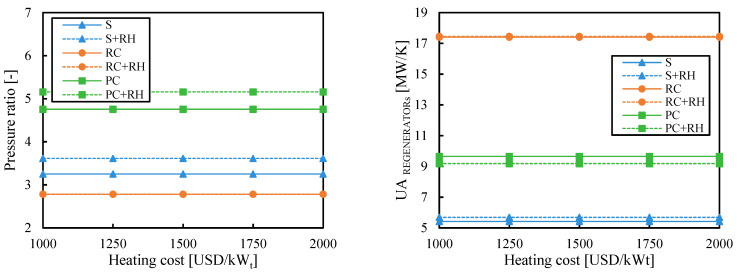
Pressure ratio and regenerator conductance used to maximize the cycle efficiency shown in Figure 9.

**Figure A6:**
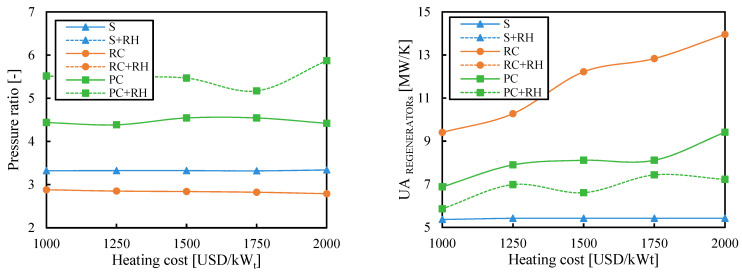
Pressure ratio and regenerator conductance used to minimize the electricity-specific cost shown in Figure 10.

**Figure A7:**
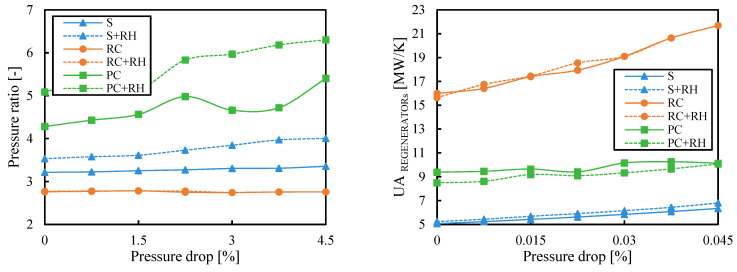
Pressure ratio and regenerator conductance used to maximize the cycle efficiency shown in Figure 11.

**Figure A8:**
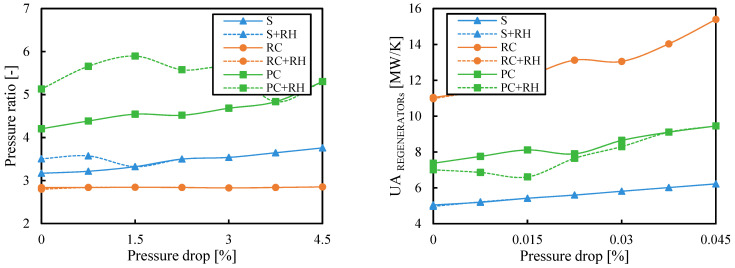
Pressure ratio and regenerator conductance used to minimize the electricity-specific cost shown in Figure 12.

**Figure A9:**
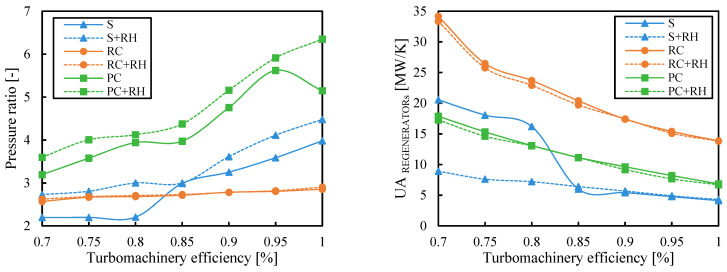
Pressure ratio and regenerator conductance used to maximize the cycle efficiency shown in Figure 13.

**Figure A10:**
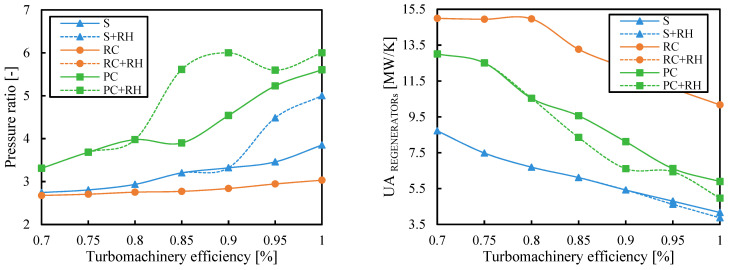
Pressure ratio and regenerator conductance used to minimize the electricity-specific cost shown in Figure 14.

## Data Availability

Data are contained within the article.

## Nomenclature

CAPEX	capital expenditure	RC	recompression cycle
EES	engineering equation solver	RC-RH	recompression cycle with reheating
HPT	high-pressure turbine	Rc	recompressor
HTR	high-temperature regenerator	REG	regenerator
IC	intercooler	RH	reheating
LCoE	levelized cost of electricity	RHX	reheater
LPT	low-pressure turbine	S	simple cycle
LTR	low-temperature regenerator	S-RH	simple cycle with reheating
MC	main compressor	sCO_2_	supercritical CO_2_
NETL	National Energy Technology Laboratory	SF	solar field
PC	partial cooling cycle	SR	split ratio
PC-RH	partial cooling cycle with reheating	T	turbine
Pc	precooler	TIT	turbine inlet temperature
PHX	primary heat exchanger	TM	turbomachinery
Pre-C	precompressor	UA	heat exchanger conductance

## Appendix A

The cost of each component is computed as [33]:

$$Cost=a\times {SP}^{b}\times f_{T}$$

where SP is the scaling parameter of each component and $f_{T}$ is its temperature correction factor:

$$f_{T}=\left\{\begin{array}{c} \;\;\;\;\;\;\;\;\;\;\;\;\;\;\;1,\;T_{max}<T_{bp}\\ 1+c\times \left(T_{max}-T_{bp}\right)+d\times {\left(T_{max}-T_{bp}\right)}^{2},\;T_{max}\geq T_{bp} \end{array}\right.$$

Being $T_{max}$ the maximum temperature the component withstands and $T_{bp}=550\;℃$ the temperature breakpoint. W stands for the work performed by each component and must be expressed in MW units. UA unit must be W/K.

**Table A1:** Cost fit coefficients for each component.

Component	a	b	c	d
PHX/RHX	3500	1	0	5.4 × 10^−5^
REG/LTR/Additional heat regenerator/HTR	49.45	0.7544	0.02141	0
Direct air coolers/IC	32.88	0.75	0	0
Axial turbines	182,600	0.5561	0	1.106 × 10^−4^
IG centrifugal MC/Rc/Pre-C	1,230,000	0.3992	0	0
Gearbox	177,200	0.2434	0	0
Generator	108,900	0.5463	0	0
Motor MC/Rc/Pre-C–open drip-proof motor	399,400	0.6062	0	0

**Table A2:** Scaling parameter for each component.

Component	SP
PHX/RHX	0
REG/LTR/Additional heat regenerator/HTR	UA
Direct air coolers/IC	UA
Axial turbines	$W_{T}$
IG centrifugal MC/Rc/Pre-C	$W_{C}$
Gearbox	$W_{T}$
Generator	$W_{E}$
Motor MC	$W_{C}$
Motor Rc	$W_{RC}$
Motor Pre-C	$W_{PCM}$

## Appendix B

The optimum values of pressure ratio and conductance obtained to maximize efficiency and minimize the electricity-specific cost can be found in this section.

### Appendix B.1. Turbine Inlet Temperature

Figure A1 shows the pressure ratio and regenerator conductance used to maximize the cycle efficiency shown in Figure 4.

Figure A2 shows the pressure ratio and regenerator conductance used to minimize the electricity-specific cost shown in Figure 5.

### Appendix B.2. Ambient Temperature

Figure A3 shows the pressure ratio and regenerator conductance used to maximize the cycle efficiency shown in Figure 7.

Figure A4 shows the pressure ratio and regenerator conductance used to minimize the electricity-specific cost shown in Figure 8.

### Appendix B.3. Heating Cost

Figure A5 shows the pressure ratio and regenerator conductance used to maximize the cycle efficiency shown in Figure 9.

Figure A6 shows the pressure ratio and regenerator conductance used to minimize the electricity-specific cost shown in Figure 10.

### Appendix B.4. Pressure Drop

Figure A7 shows the pressure ratio and regenerator conductance used to maximize the cycle efficiency shown in Figure 11.

Figure A8 shows the pressure ratio and regenerator conductance used to minimize the electricity-specific cost shown in Figure 12.

### Appendix B.5. Turbomachinery Efficiency

Figure A9 shows the pressure ratio and regenerator conductance used to maximize the cycle efficiency shown in Figure 13.

Figure A10 shows the pressure ratio and regenerator conductance used to minimize the electricity-specific cost shown in Figure 14.
